# Continuous erector spinae plane block versus intercostal nerve block in patients undergoing video-assisted thoracoscopic surgery: a pilot randomized controlled trial

**DOI:** 10.1186/s40814-021-00801-7

**Published:** 2021-02-24

**Authors:** Dillon Horth, William Sanh, Peter Moisiuk, Turlough O’Hare, Yaron Shargall, Christian Finley, Waël Hanna, John Agzarian, Mauricio Forero, Kim Davis, Thuva Vanniyasingam, Lehana Thabane, Harsha Shanthanna

**Affiliations:** 1 Department of Anesthesia, McMaster University, Hamilton, Ontario Canada; 2 Department of Surgery, McMaster University, Hamilton, Ontario Canada; 3 Department of Anesthesia, St. Joseph’s Health Care Hamilton, Hamilton, Ontario Canada; 4 Health Research Methods, Evidence and Impact, McMaster University, Hamilton, Ontario Canada

**Keywords:** Erector spinae plane block, Intercostal nerve blockade, Perioperative analgesia, Video-assisted thoracoscopic surgery

## Abstract

**Background:**

The optimal analgesia method in video-assisted thoracoscopic surgery (VATS) remains controversial. Intercostal nerve blockade (ICNB) is limited by its duration of action. The erector spinae plane (ESP) block has the potential to provide satisfactory analgesia for VATS; however, the effectiveness of continuous ESP versus surgeon-performed ICNB has not been investigated. The objectives of this study were to establish feasibility of patient recruitment and follow-up before undertaking a fully powered randomized controlled trial (RCT); and, secondarily, to compare opioid usage, pain control, and sensory blockade.

**Methods:**

This feasibility RCT was undertaken at St Joseph’s Hospital, Hamilton, Ontario, Canada, and included 24 patients (>18 years) having elective VATS with at least one overnight stay. Exclusion criteria were patient refusal, body mass index >40 kg/m^2^, contraindications to neuraxial analgesia techniques as per the American Society of Regional Anesthesia and Pain guidelines, known allergy to local anesthetics, language or comprehension barriers, procedures with a higher chance of open surgery, and regular opioid use for ≥3 months preoperatively. Patients underwent either continuous ESP (*n*=12) or surgeon-performed ICNB (*n*=12). All patients received routine intraoperative anesthesia care and multimodal analgesia. Feasibility criteria were recruitment rate of two patients/week and full follow-up in all patients in-hospital. We compared opioid consumption, postoperative pain scores (0–10 numerical rating scale), adverse events, patient satisfaction, and distribution of sensory blockade as clinical outcomes (secondary).

**Results:**

Feasibility of primary outcomes was successfully demonstrated. Five patients had an epidural in anticipation of open surgery. Mean opioid consumption as equivalent morphine units was less in the ESP group over the first 24 h (mean difference, 1.63 [95% CI –1.20 to 4.45]) and 48 h (mean difference, 2.34 [95% CI –1.93 to 6.61]). There were no differences in adverse effects.

**Conclusions:**

A fully powered RCT is feasible with modifications. Our results also suggest that continuous ESP is safe and can decrease opioid needs. However, it is important to consider procedures to improve compliance to protocol and adherence to assigned interventions.

**Trial registration:**

Clinicaltrials.gov identifier: NCT03176667. Registered June 5, 2017.

**Supplementary Information:**

The online version contains supplementary material available at 10.1186/s40814-021-00801-7.

## Introduction

Video-assisted thoracoscopic surgery (VATS) is an increasingly popular technique in thoracic surgery with improvements in technology. VATS provides significant advantages over open thoracotomy procedures including reduced surgical pain, reduced mortality, improved postoperative pulmonary function, and shorter hospital length of stay [1–4]. Nevertheless, there is still considerable amount of postoperative acute pain with VATS lobectomies. Controlling postoperative pain is crucial because increased acute pain has been associated with the development of chronic pain [5], increased hospital length of stay [6], and decreased patient satisfaction [7].

Most patients receive thoracic epidural analgesia (TEA) to treat postoperative pain in thoracotomy procedures [8]. For VATS procedures, the merits of these regional techniques are uncertain as compared with patient-controlled analgesia (PCA) with opioids [9–11]. The paravertebral block (PVB) is an alternative to TEA; however, both TEA and PVB have the potential for serious side effects and complications including severe hypotension, epidural bleeding, and spinal cord or nerve injury. Although PVB is associated with a lower incidence of hypotension, it carries the risk of pneumothorax, pleural and vascular puncture, and higher systemic absorption of local anesthetics. PVB is also technically challenging and does not always reliably spread in the paravertebral space [12]. In addition, placement of both TEA and PVB requires appropriate discontinuation of anticoagulants. Intercostal nerve blocks (ICNBs) have been used as an analgesic alternative to TEA and PVB in VATS with beneficial effect [13, 14]. However, although ICNBs have shown to lower pain scores in the early postoperative period [15], they have a limited duration of effect [16]. A novel regional technique, the erector spinae plane (ESP) block, has been recently described [17]. This interfascial block involves ultrasound (US)-guided injection of local anesthetics posterior to the erector spinae muscle and superficial to the transverse process of thoracic vertebrae at the appropriate level. The ESP block predominantly targets the dorsal rami of the spinal nerves as they leave the intervertebral foramen, but also has the potential to block ventral rami [18]. Cadaveric examination of ESP block showed extensive cranial-caudal spread of the block, approximately four dermatomes above and below the site of injection. The successful use of the ESP block has led to studies in multiple clinical settings including abdominal surgery [17], VATS [18], and cardiac surgery [19]. The simplicity and safety of the ESP block has been proposed as its main advantages. Potentially, a catheter placed at the ESP plane allows continuous infusion and prolonged analgesia [19].

Given the importance of providing adequate analgesia in VATS procedures and lack of consensus among surgeons and anesthesiologists for the optimal analgesic technique, we planned to conduct a randomized controlled trial (RCT) to compare continuous ESP blockade versus surgeon-performed ICNBs in patients receiving VATS lobectomy or wedge resections, with the primary objective of assessing the difference in opioid analgesia used. Before conducting the main trial, we conducted a pilot RCT, with the primary objective of establishing feasibility of patient recruitment and follow-up.

## Methods

This was a two-arm randomized controlled feasibility trial conducted at St Joseph’s Hospital, Hamilton, Ontario, Canada. The study was registered at clinicaltrials.gov with the registration number NCT03176667, and ethics approval was obtained from the Hamilton Integrated Research Ethics Board on July 18, 2017 (#3012). Due to the unavailability of rupivacaine at the start of the study, as well as input regarding epinephrine from participating anesthesiologists, patients undergoing ESP were administered 30 ml of 0.125% bupivacaine rather than 30 ml of 0.5% ropivacaine with 5 μg ml^-1^ of epinephrine. Patient enrollment began December 2018.

### Participants

Patients were screened for trial inclusion using the preoperative booking list. Adult patients (>18 years) having elective unilateral VATS procedures with at least one overnight stay were included. Patients were excluded if they had one or more of the following: patient refusal, body mass index >40 kg m^-2^, and contraindications to neuraxial analgesia techniques as per the American Society of Regional Anesthesia and Pain guidelines [20–22]; known allergy to local anesthetics; inability to use PCA due to language or comprehension barriers; procedures with a higher chance of open surgery; and patient on any regular opioids for ≥3 months prior to surgery. During their pre-operative visit, a research assistant approached suitable patients, explained study procedures, and answered their questions. Patients had the opportunity to withdraw at any point. Baseline data, including demographics and history of comorbidities were collected at the same visit.

### Randomization, allocation, and blinding

Patients were randomized to intervention (US-guided continuous ESP block) and control (surgeon-performed ICNB) groups with a 1:1 allocation ratio using a computerized randomization the day before surgery. Randomization was generated by a statistician not involved with the study, and the list was provided to the pharmacy. The group allocation was concealed and revealed to the anesthesiologist assigned to manage the surgical case the day before to ensure that a trained anesthesiologist was available to perform the pre-operative ESP procedure and catheter placement. Due to the nature of the study interventions, patients and research personnel were not blinded.

### Intervention group

In the intervention group, patients had a pre-operatively performed US-guided ESP block with catheter placement in a designated block room equipped with monitoring and resuscitation facilities. Intravenous (i.v.) access was established and standard American Society of Anesthesiologists monitors were applied. Sedation and anxiolytics were used as considered appropriate. Patients were placed in a sitting position and the area was sterilized with disposable swabs of 2% chlorhexidine in 70% isopropyl alcohol and then draped in a sterile fashion. A high-frequency linear US transducer (GE LOGIQe, Wauwatosa, WI, USA) was used in a longitudinal parasagittal orientation 3 cm lateral to the T5 spinous process. The trapezius, rhomboid major, and erector spinae muscles were identified superficial to the tip of the T5 transverse process. The patient’s skin was anesthetized with 2% lidocaine. A B-Braun Contiplex Echo 18 gauge Tuohy needle with the Contiplex Echo 20 gauge catheter (Braun Medical Inc., Bethlehem, PA, USA) was inserted using an in-plane superior-to-inferior approach to place the tip into the fascial plane on the deep (anterior) aspect of the erector spinae muscle. The location of the needle tip was confirmed by visible fluid spread lifting the erector spinae muscle off the bony shadow of the transverse process. A maximum of 30 ml of 0.125% bupivacaine was injected in 5-ml aliquots through the needle (maximum of 3 mg kg^-1^) followed by insertion of a 19 gauge catheter under direct vision 5 cm beyond the needle tip.

### Control group

In the control group, the thoracic surgeons performed ICNBs from T4 to T11 on the operated side of the chest, at the end of surgical procedure. The ICNBs were performed using 0.25% bupivacaine with epinephrine and a volume of 5 ml per block (maximum of 2.5 mg kg^-1^) was used.

### Anesthesia management

The attending anesthesiologist provided a general anesthetic as per the routine institutional practice. For patients in the ESP block group, the attending anesthesiologist started a background infusion of bupivacaine 0.125% at 13 ml h^-1^ through the catheter after incision. At the end of the case a bolus of 0.125% bupivacaine 5 ml was injected through the ESP catheter and the patient was extubated. Patients in the control group had ICNBs placed at the end of surgery, then extubated. PCA was initiated postoperatively in the recovery room.

### Postoperative management

All patients were monitored in the post-anesthetic care unit (PACU) for stabilization as routine practice. If the patient complained of incisional pain of intensity of >4/10 on the numerical rating scale (NRS), the nurse provided boluses of hydromorphone 0.2–0.4 mg i.v. every 7 min as needed to a maximum of 2 mg, after which PCA pumps were initiated. If the patient was allergic to hydromorphone, a morphine PCA was substituted. All patients were enrolled into the Acute Pain Service (APS) and were visited daily by a nurse practitioner and/or an anesthesiologist. In the ESP group, continuous ESP block was provided with a background infusion of 0.125% bupivacaine at 13 ml h^-1^.

The PCA pump was programmed to administer hydromorphone in boluses of 0.2 to 0.4 mg i.v., with lockout time of 7 to 10 min to a maximum 6 mg over 4 h; or morphine in boluses of 1 to 2 mg i.v., with a lockout time of 7 to 10 min to a maximum of 30 mg over 4 h. Changes to PCA opioid doses was made by the APS team, as necessitated for each patient to target minimal or tolerable pain (NRS <4). In both groups, patients had multimodal analgesia, individualized based on the allergies, comorbidities, and patient tolerance: (1) acetaminophen 975 mg orally/per rectum (PO/PR) for 48 h followed by 650 mg PO/PR every 4 h as needed until discharge and (2) naproxen 500 mg PO twice daily × eight doses (administer with food) or ketorolac 10 mg i.v. four times daily × eight doses if patient is unable to ingest PO medications. PCA was discontinued upon removal of the chest tubes or when patient was ready for discharge, as is the current standard of practice at our hospital. ESP catheter infusion was discontinued 48 h after surgery or under any one or more of the following circumstances: chest drain removal, patient discharge, any signs of local infection or systemic infection, or any sign of mechanical dysfunction including leaking or inadvertent withdrawal of the catheter.

### Study outcomes

#### Primary outcomes (feasibility)

To assess the feasibility of conducting a large RCT comparing the ESP versus ICNB, the following outcomes were evaluated: (1) recruitment rate (number of participants recruited per week and (2) proportion of in-hospital follow-up until discharge. The study was considered feasible if the recruitment rate was greater than two patients per week and with complete (100%) in-hospital follow-up until discharge for the clinical outcomes.

#### Secondary outcomes (clinical)

Among the following clinical outcomes, differences in total opioid consumed over the first 24 h and 48 h were explored as primary outcomes for the larger RCT. We considered evaluating opioid use at both 24 and 48 h separately since a substantial percentage of patients were being discharged before 48 h. For the main study, we plan to consider only one of the two as our primary outcome.

1. Differences in total opioid consumption over the first 24 h and 48 h. This was collected using electronic medical records. Opioids will be considered as total hydromorphone equivalents for analysis.
2. Difference in pain scores “at rest” and “at movement” at the following time points: 1 h after entry at PACU, average pain score during the postoperative nights as collected during the morning APS rounds, and pain scores on each morning until discharge. Pain scores at movement were collected by asking patients to cough. Both pain scores were recorded using the patient-reported NRS, an 11-point scale where 0 is no pain and 10 is the worst pain imaginable (Additional file 1: Appendix 1). The NRS is validated and considered easy to use [23].
3. Adverse effects: Difference in the incidence of postoperative nausea-vomiting, respiratory depression, itching, local anesthetic toxicity, catheter leakage and catheter migration, and infection around the catheter site. The definitions and scales of each of the outcomes are provided in Additional file 1: Appendix 1.
4. Presence of sensory blockade on the operated side of the chest was collected on the first postoperative morning. Sensory assessment was performed by trained data collectors for pin prick (using a blunt needle). The dermatome distribution of the blockade on the patient’s anterior chest, mid-axillary line, mid-clavicular line, and mid-scapular line was assessed using a blunt needle to test for loss of sensation to pinprick. Sensory testing was performed and recorded as shown in Additional file 1: Appendix 1.
5. Patient satisfaction with postoperative analgesia on the day of discharge. This was performed using a five-point Likert scale (Additional file 1: Appendix 1).

### Statistical analysis and sample size

The analysis and reporting of the trial was performed in accordance with the Consolidated Standards of Reporting Trials (CONSORT) guidelines for pilot studies [24]. Baseline and intraoperative data were reported using means and standard deviations or median and interquartile range as appropriate. We analyzed as intent to treat (ITT) with each patient analyzed according to the group to which they were randomized. Clinical outcomes were compared using a two-sided test with a significance level of 0.05. No subgroup tests were conducted. As suggested in literature for pilot studies, we considered a sample size of 12 patients per group to demonstrate feasibility [24, 25].

### Data collection and confidentiality

Patients were noted using a study ID to keep their information anonymous. The patient’s study ID and corresponding hospital medical record number were recorded in an Excel file in a password-encrypted USB that was securely stored in a locked cabinet. Data collection was done using paper forms and transferred to REDCap for secure storage and analysis. Data were collected by research assistants and the APS nurse practitioner (KD). The data collection forms were also kept in the locked cabinet.

## Results

**Fig. 1:**
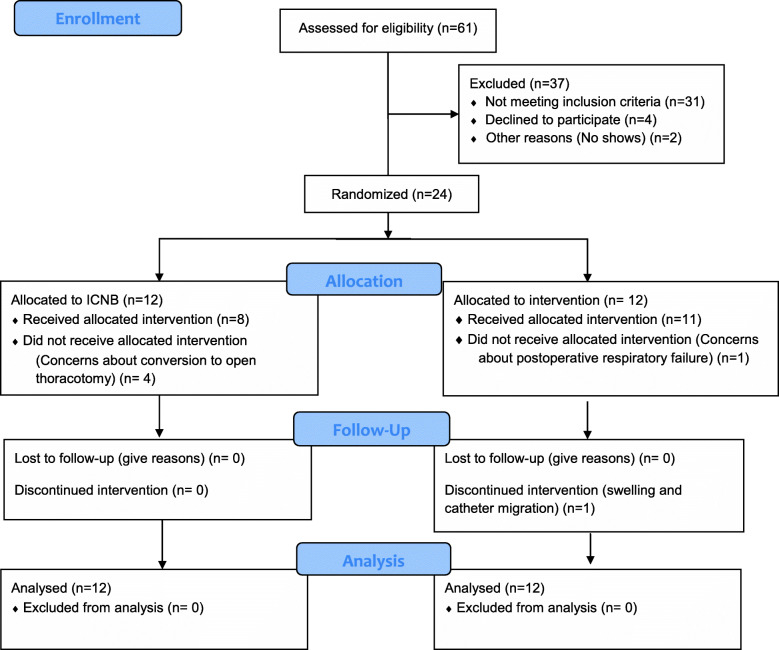
Consort flow diagram

The study was carried out from November 2018 to February 2019. Of the 54 patients meeting eligibility criteria 24 patients were consented and randomized to the intervention (*n*=12) and control (*n*=12) groups (Fig. 1). Table 1 and Table 2 list the baseline and intraoperative characteristics, respectively. The most common procedure performed was VATS lobectomy (50%), followed by thoracoscopic wedge resections (42%). Two cases (8%; both from the intervention group) were converted to open lobectomy. The rate of adherence to the assigned treatment was 92% in the intervention group and 67% in the control group. Epidural catheters were inserted in five patients; four with a higher chance of converting to open surgeries intraoperatively and one patient about whom the treating anesthesiologist had concerns about postoperative ventilatory failure based on predicted forced expiratory volume in 1 s.

**Table 1:** Baseline characteristics

Baseline characteristics	Control (*n*=12)	Intervention (*n*=12)
Age, years; mean ± SD	66.50 ± 8.44	71.33 ± 9.00
Females; *n* (%)	10 (83.33)	7 (58.33)
BMI, kg m^-2^; mean ± SD	25.10 ± 5.16	28.34 ± 4.03
Weight, kg; mean ± SD	66.37 ± 20.14	77.81 ± 13.21
ASA 3^a^; *n* (%)	7 (58.33)	7 (58.33)
ASA 4^a^; *n* (%)	5 (41.67)	5 (41.67)

^a^There were no participants belonging to ASA categories of 1, 2, or 5. *ASA* American Society of Anesthesiologists, *BMI* body mass index

**Table 2:** Intraoperative characteristics

Intraoperative characteristics	Control (*n*=12)	Intervention (*n*=12)
Diagnosis; *n* (%)		
Lung cancer	8 (66.7)	9 (75)
Lung nodule	4 (33.3)	3 (25)
Type of surgery; *n* (%)		
VATS lobectomy	7 (58)	5 (42)
Thoracoscopic wedge resection	5 (42)	5 (42)
Converted open lobectomy	0	2 (16)
Type of analgesia; *n* (%)		
Epidural	4 (33.3)	1 (8)
Adherence to assigned analgesia modality; *n* (%)	8 (66.7)^a^	11 (91.7)^a^

^a^Individuals who did not receive the correct assigned treatment (ESP or ICNB) received a thoracic epidural. *ESP* erector spinae plane block, *ICNB* intercostal nerve block

### Primary outcomes

We were successful in demonstrating feasibility for both of our primary outcomes. As the recruitment happened over 58 days (11.6 weeks), the number of patients recruited per week was >2 and we successfully followed all patients for clinical outcomes during their hospital stay.

### Clinical outcomes

**Fig. 2:**
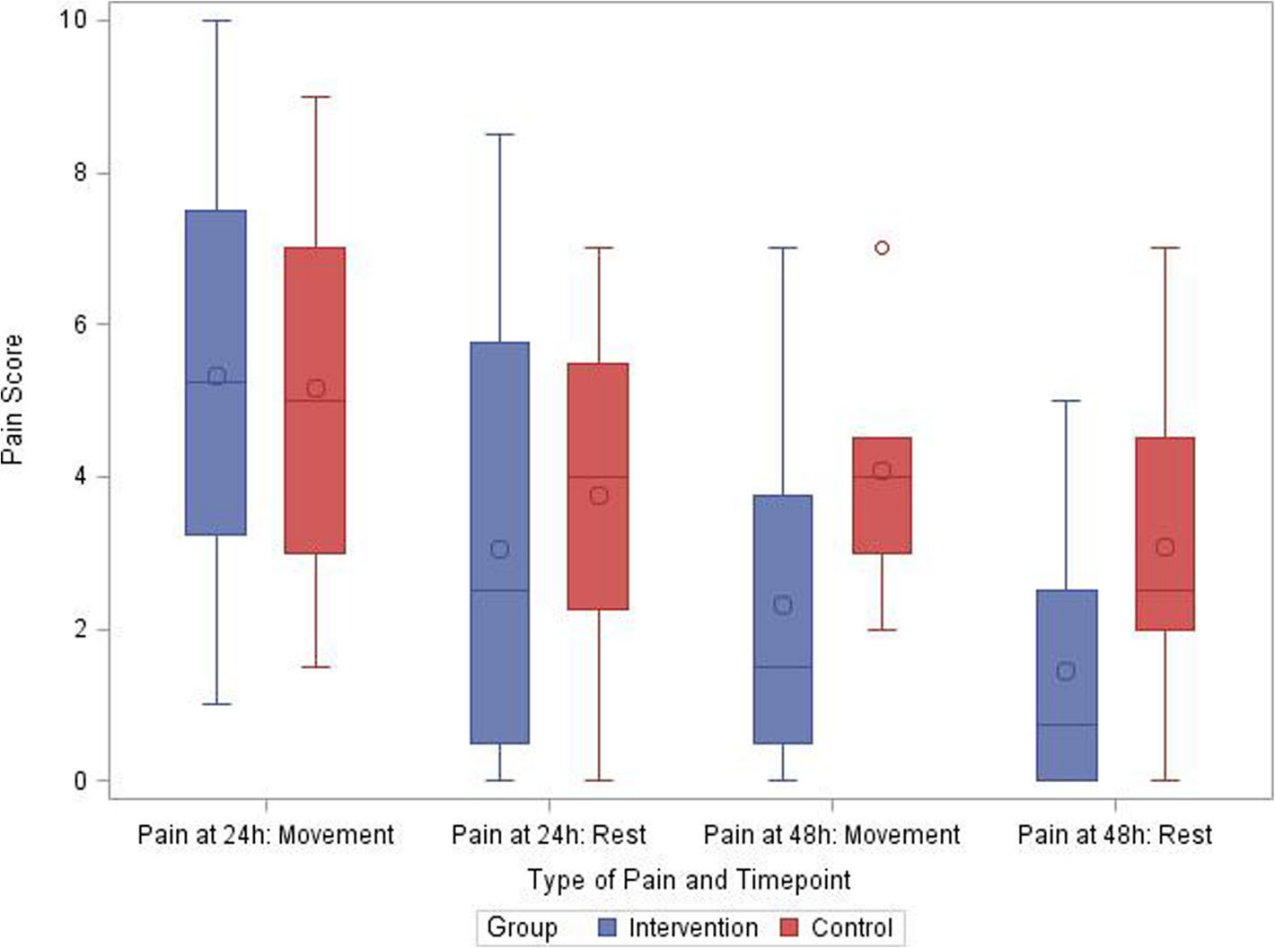
Pain scores (box and whisker plot)

**Fig. 3:**
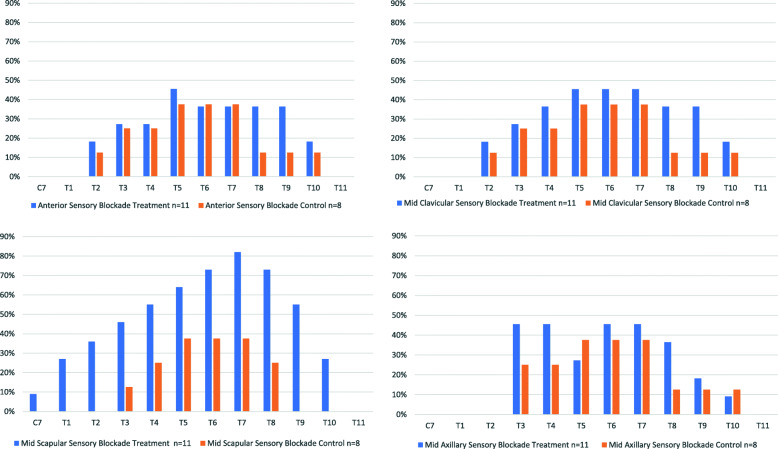
Comparison of sensory blockade. **a** Sensory blockade at the anterior chest. **b** Sensory blockade at the mid-clavicular level. **c** Sensory blockade at the mid-scapular level. **d** Sensory blockade at the mid-axillary level

**Fig. 4:**
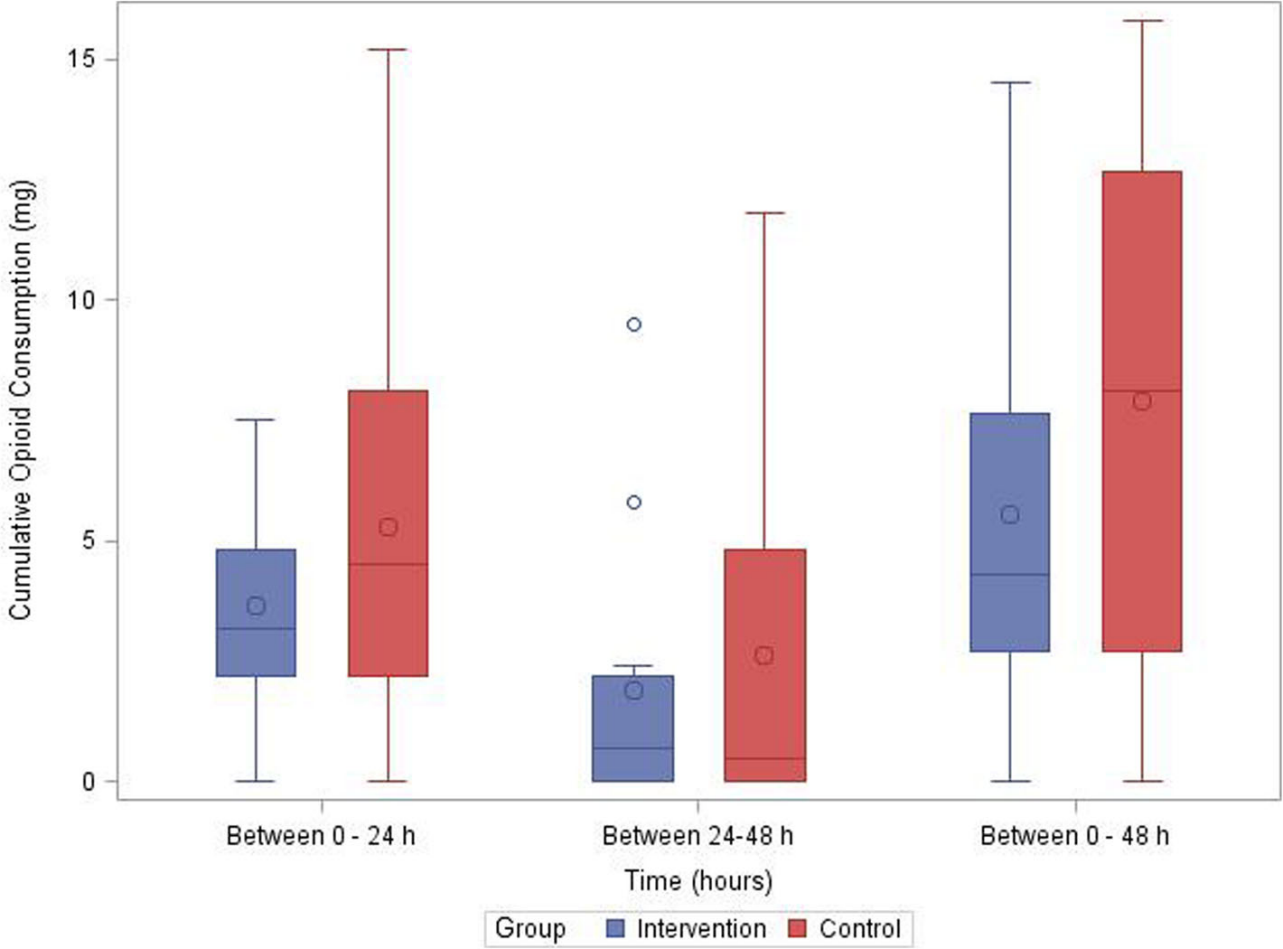
Cumulative opioid consumption (box and whisker plot)

Clinical outcomes are reported in Table 3 and Figs. 2, 3, and 4. As a feasibility trial, the trial was not designed to test for statistical significance within these outcomes. The mean opioid consumption was less in the intervention group over the first 24 h; mean difference (MD) of 1.63 (95% confidence interval [CI] –1.20 to 4.45), and 48 h, MD of 2.34 (95% CI –1.93 to 6.61) (Table 3, Fig. 4). Average pain on the first postoperative morning (which consisted of the first postoperative evening pain score and first postoperative morning pain score) was recorded in all patients and showed minimal difference between the groups at rest: MD 0.71 (95% CI –1.47 to 2.88); and with movement: MD –0.17 (95% CI –2.49 to 2.16). Before the second postoperative morning 10 study, patients had their chest tubes and their continuous analgesia (PCA and ESP infusion) removed. Hence, we only had 14 patients reporting their pain scores on the second postoperative morning. The pain scores (which consisted of the second postoperative day evening pain score and second postoperative day morning pain score) were less in the intervention group at rest: MD 1.65 (95% CI –0.89 to 4.19); and with movement: MD 1.77 (95% CI –0.86 to 4.41).

**Table 3:** Summary of secondary outcomes

Outcome	Control	Treatment	MD (95% CI)
Cumulative opioid consumption (mg) at 24 h, mean ± SD	5.28 ± 4.17 *n*=12	3.66 ± 2.22 *n*=12	1.63 (–1.20 to 4.45)
Cumulative opioid consumption (mg) between 24–48 h, mean ± SD	2.61 ± 3.77 *n*=12	1.89 ± 2.91 *n*=12	0.72 (–2.14 to 3.57)
Cumulative opioid consumption (mg), at 48 h, mean ± SD	7.89 ± 5.58	5.55 ± 4.44	2.34 (–1.93 to 6.61)
Pain score at rest^a^			
Average pain after 24 h, mean ± SD	3.75 ± 2.18 *n*=12	3.04 ± 2.90 *n*=12	0.71 (–1.47 to 2.88)
Average pain after 48 hr, mean ± SD	3.08 ± 2.42 *n*=6	1.44 ± 1.95 *n*=8	1.65 (–0.89 to 4.19)
Pain scores with movement^a^			
Average pain after 24 h; mean ± SD	5.17 ± 2.61 *n*=12	5.33 ± 2.88 *n*=12	–0.17 (–2.49 to 2.16)
Average pain after 48 h, mean ± SD	4.08 ± 1.69 *n*=6	2.31 ± 2.56 *n*=8	1.77 (–0.86 to 4.41)
Adverse event: Catheter migration	0	1	
Adverse event: Catheter disconnection	0	1	
Patient satisfaction, *n* (%)			
Very satisfied	7 (58.33)	8 (66.67)	
Satisfied	3 (25.00)	3 (25.00)	
Neutral	2 (16.67)	1 (8.33)	
Required treatment for nausea and vomiting in PACU, *n* (%)	1 (8.33)	2 (16.67)	.
Required treatment for nausea and vomiting in the first 24 h postoperatively (excluding PACU), *n* (%)	6 (50)	3 (25)	.

^a^Numerical rating scale, an 11-point scale where 0 is no pain and 10 is the worst pain imaginable. *MD* mean difference, *PACU* post-anesthetic care unit

Two individuals (both in the intervention group) experienced at least one adverse event: one event was catheter migration at 24 h, and the second was an accidental catheter disconnection. Nine individuals needed treatment for nausea and vomiting within 24 h, in which three also required treatment in PACU. Among the five categories of patient satisfaction, there were no patients in either group that recorded dissatisfied or completely dissatisfied. In the control group, 58% were very satisfied, 25% were satisfied, and 17% were neutral. In the treatment group, 67% were very satisfied, 25% were satisfied, and 8% were neutral.

The presence of sensory blockade on the operative side of the thorax in each group is shown according to dermatome in Fig. 3. Figure 3a indicates that the difference between the two groups was more obvious at the mid-scapular line compared with other areas. Within the intervention group, peak blockade was observed at dermatomes T5, T6, and T7 for most areas and the percentage of patients who had such blockade were less than 50% of patients, except at the mid-scapular line.

## Discussion

In this pilot trial, we were able to demonstrate the feasibility of conducting an RCT comparing continuous ESP analgesia versus ICNB in patients undergoing VATS. We were able to recruit more than two patients per week and follow all patients during their hospital stay. We also observed that the study intervention had a positive influence on the opioid consumption and postoperative pain scores. As a feasibility trial, it was not powered to assess for clinical outcomes. There were no serious adverse clinical events that posed a threat to patient safety or resulted in any known morbidity or mortality. Based on our pilot study, and using the same comparators, we would need a total of 63 patients/group for a difference of 1.63 mg between the groups. We provide estimates of sample size with other variations to mean difference and SDs of the treatment and comparator group in our supplemental table (Additional file 2: Appendix 2).

A few other trials have explored the effectiveness of single-shot ESP blocks in VATS and thoracotomies. However, not many studies have assessed the value of continuous ESP block for VATS patients. Fang et al. [26] demonstrated that, compared with a single PVB, a single-shot ESP block with 20 ml of 0.25% bupivacaine provided no differences in pain scores and postoperative opioid consumption in patients undergoing thoracotomies, with less incidence of hypotension. Chen et al. [27] compared ICNB, a single-shot ESP block, and multiple paravertebral nerve blocks for VATS. They found that PVBs provided superior analgesia with reduction in visual analog scale scores and opioid consumption in comparison to both single-shot ESP blocks and intercostal blocks. However, when single-shot ESP blocks and intercostal blocks were compared with one another they were equally effective in reducing pain. Gaballah et al. [28] reported that patients treated with a single ESP block had lower visual analogue scale scores and longer time to first analgesia compared with patients who received a single shot of serratus plane block. The results of our study suggest similar findings to these studies, showing that a continuous ESP block may provide superior analgesia through decreased pain scores and decreased opioid consumption compared with a single-shot intercostal block. A recent trial observed that analgesia provided by ESP was non-inferior to PVB for VATS resections, and ESP also resulted in lower plasma concentrations of levobupivacaine [29]. This further substantiates the relative safety of ESP over PVB.

One important observation that warrants attention and planning prior to a larger scale RCT using our study design is how to optimize adherence to the assigned interventions. As noted above, five of the 24 patients received a thoracic epidural and four in the control group and one in the intervention group. In four patients, epidurals were inserted in consideration of a higher chance of converting to open surgeries intraoperatively, but only two patients were actually converted to thoracotomy. Our trial was not blinded to patients and healthcare providers and there may be a bias toward considering an epidural in the control group for patients with a higher chance of open conversion. Because continuous epidural is a potent intervention for pain relief and reducing opioids, it has the potential to influence clinical outcomes for the group. It is important to find ways to improve adherence as it may increase the chances of type II error (false negative). Possible solutions include reminding physicians that all procedures have a risk of being converted to open thoracotomy, and routinely placed thoracic epidurals are not currently the standard of practice at our institution. Furthermore, thoracic epidurals may also be placed postoperatively if indicated. Tightening the selection criteria so that only suitable patients are randomized can be considered. However, tightening the selection criteria has important implications to study recruitment. There have been suggestions that ITT analysis may not always be appropriate in circumstances such as the above where inappropriate patients were included, and it might be better to go with the recommendations of an adjudication committee for each case inclusion. Finally, another point that should be mentioned is the option to exclude any case that gets converted to open thoracotomy.

It has been observed in the literature that the ESP block has variable dermatomal spread. We note that in comparison to ICNB from T4-T8 placed by the surgeon, an ESP placed at T5 covered more dermatomes in our study. Other reports have shown that ESP has the potential for a wider spread. It is probably likely that more obvious blockade at the mid scapular line is explained by predominant blockade of posterior branches. Using computed tomography with contrast, Forero et al. [17] demonstrated that injection of 20 ml of 0.5% bupivacaine at T5 showed spread between T1 and T11 vertebra. Scimia et al. [30] similarly showed that a continuous ESP block at T6 in a patient receiving VATS lobectomy had T2–T10 dermatomal spread using a cold test in the postoperative period.

We believe our primary clinical outcome of reduction in opioid usage is more appropriate than pain scores. Since opioid usage was based on PCA needs, it still reflects pain perceived by a patient indirectly if one assumes that opioid needs are based on individual pain tolerance. Opioid usage was observed to be lower in the intervention arm at all points. The decrease in opioid consumption may also explain the decrease in anti-emetic drug administration seen in the ESP and PCA arm. Although we did not intend to limit the inclusion based on the ASA score, all of our patients belonged to either ASA 3 or 4 risk category. We recognize that this might potentially affect the external validity of the study results and hence we plan to ensure inclusion of a wider range of ASA score patients for our main trial.

There are a few important limitations of our study. This pilot RCT was not blinded; hence, it carries a higher potential for observation bias. It should also be emphasized that this is a pilot study and it was not designed or powered to provide clinical inference. As mentioned above, there were five patients that had an epidural, indicating non-compliance with study protocol. In view of this, we could consider patient selection criteria as a limitation that needs to be improved in our main trial.

## Conclusions

In conclusion, we conducted a pilot, non-blinded RCT comparing continuous ESP blockade with surgeon-performed ICNB in patients having VATS resections. Our trial demonstrated that it would be feasible with modifications to conduct a fully powered RCT. We further demonstrate that this type of trial is safe and that there are indications of lower opioid consumption and superior analgesia in the ESP arm. However, more research is required to determine whether this effect is clinically meaningful and statistically significant.

## Supplementary Information

**Additional file 1: Appendix 1** A supplementary document that contains definitions and scales of each outcome, and diagrams of sensory testing.

**Additional file 2: Appendix 2** Appendix_Calculation of group sample size. A supplementary document that presents the data for sample size estimates for the main trial (primary outcome, cumulative opioid consumption at 24 h).

## Data Availability

Data are available from the corresponding author upon request.

## Abbreviations

APS: Acute Pain Service
ASA: American Society of Anesthesiologists
BMI: Body mass index
CI: Confidence interval
CONSORT: Consolidated Standards of Reporting Trials
ESP: Erector spinae plane block
ICNB: Intercostal nerve blockade
ITT: Intent to treat
i.v.: Intravenous
MD: Mean difference
PCA: Patient-controlled analgesia
PO: Orally
PR: Per rectum
PVB: Paravertebral block
RCT: Randomized controlled trial
TEA: Thoracic epidural analgesia
US: Ultrasound
VATS: Video-assisted thoracoscopic surgery
