# Supplementary material for: Continuous erector spinae plane block versus intercostal nerve block in patients undergoing video-assisted thoracoscopic surgery: a pilot randomized controlled trial
Source: Pilot Feasibility Stud. 2021 Feb 24;7:56. doi: 10.1186/s40814-021-00801-7 (PMC7903734; doi:10.1186/s40814-021-00801-7)
Supplement: Supplementary file 1 — Additional file 1: Appendix 1 A supplementary document that contains definitions and scales of each outcome, and diagrams of sensory testing. [file 40814_2021_801_MOESM1_ESM.doc]

**APPENDIX**

1. Numerical Rating Score (NRS) 0 to 10.^1^


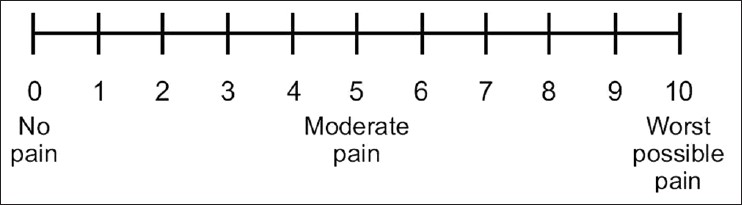


1. Nausea-Vomiting: Incidence of moderate to severe nausea-vomiting scored as 0-3 scale
2. (below), adapted from the symptom severity scale.^2^ It will be collected at PACU, over 24 hours and over 48 hours.

0-No

1-Mild

2-Moderate

3-Severe

1. Itching (how to measure): Incidence of moderate to severe itching will be measured and compared using a 0-3 scale, adapted from the symptom severity scale [2].

0-No itching

1-Mild

2-Moderate –requiring treatment

3-Severe- multiple episodes with no effect of treatment

1. Respiratory Depression – PCA pump was discontinued by the Acute Pain Service due to concerns of respiratory depression by nursing staff or respiratory rate less than 8.
2. Local anesthetic toxicity: Clinical symptoms and signs of local anesthetic toxicity. Incidence will be compared between the two groups.
3. Catheter leakage: Excessive local anesthetic leaking from site of catheter insertion prompting the nurse to change the dressing in less than 2 hours and prompting the concern of the APS team to evaluate leakage.
4. Catheter migration: catheter leakage with no sensory block or catheter completely outside the body.
5. Infection around the site of catheter site: swelling and redness observed by APS team or the nurse attending to the patient.
6. Patient Satisfaction with postoperative analgesia

1-Very Satisfied

2-Satisfied

3-Neutral

4-Dissatisfied

5-Completely Dissatisfied

1. Sensory Examination

**Post block findings: (in PACU and one 1^st^ postoperative day morning, using blunt needle)** Time:

|  | Left Upper dermatome level | Left Lower  dermatome level | Right Upper  dermatome level | Right Lower  dermatome level |
| --- | --- | --- | --- | --- |
| Posterior (mid- scapular line) |  |  |  |  |
| Mid-axillary line |  |  |  |  |
| Mid-clavicular line |  |  |  |  |
| Anterior (3 cm away from sternum) |  |  |  |  |

Sensory block


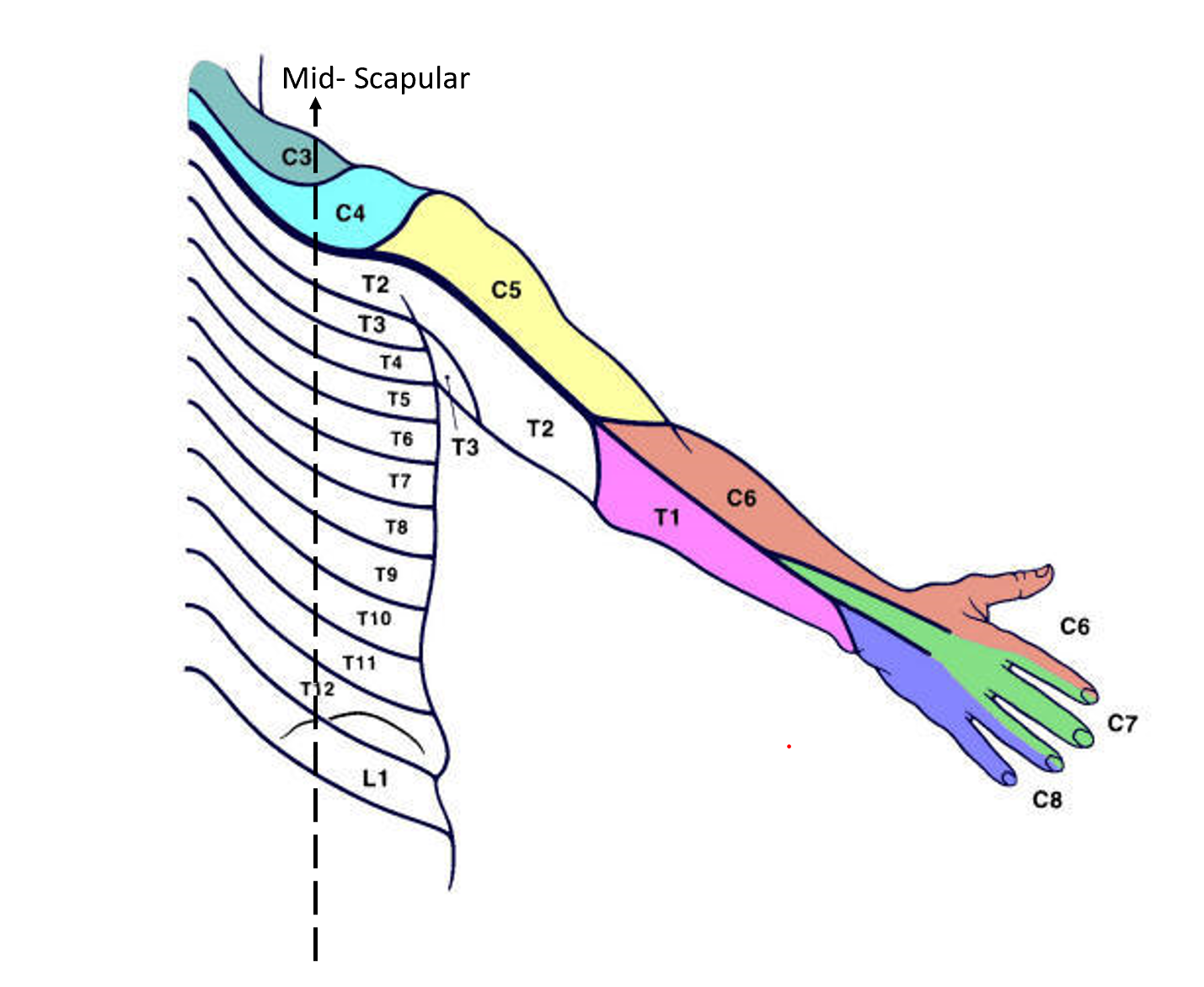


**
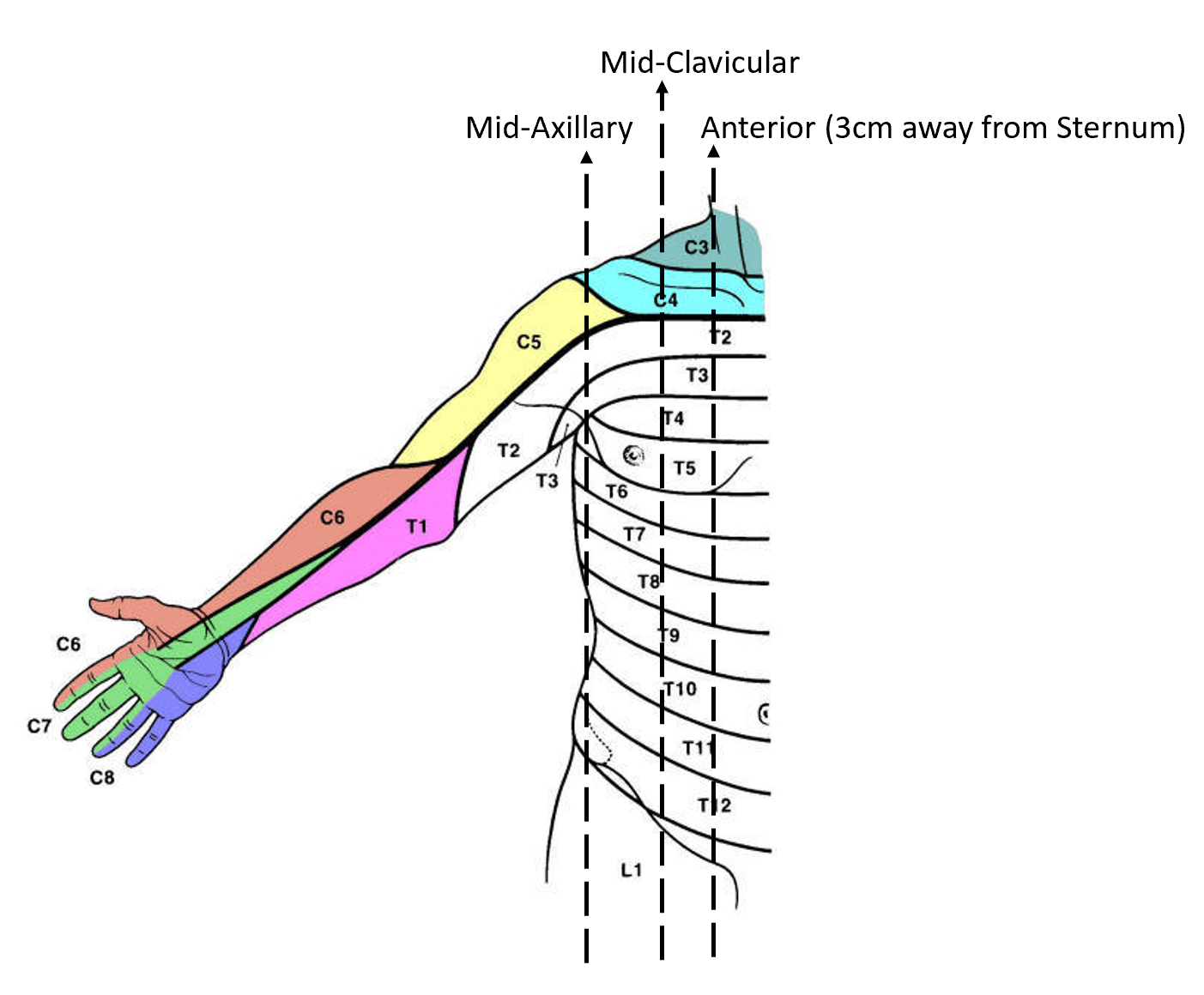
**

**List of Variables for Data Collection**

**Preoperative Variables**

| **Variable:** | **Unit:** |
| --- | --- |
| Age | Years |
| Weight | Kg |
| Height | Cm |
| BMI | Kg/m^2^ |
| ASA | 1-5 |
| Co-Existing Illnesses | Free text |
| Imaging | Free text |
| Preoperative Pain | Location (dermatomes) |
| Preoperative Pain | Duration (months) |
| Preoperative Pain | NRS (0-10) |
| Pain Medications | Free Text |

ASA: American Society of Anesthesiologists; BMI: body mass index; NRS: numerical rating scale

**Intraoperative Variables**

| **Variable**: | **Unit:** |
| --- | --- |
| Diagnosis | Free text |
| Procedure Done | Free text |
| Incision Level | Dermatome |
| Drain | Dermatome |

**Postoperative Outcomes**

| **Variable:** | **Unit:** |
| --- | --- |
| Postoperative Block Sensory Findings (posterior, mid axillary line, mid clavicular line, anterior) | Present or Absent, and Upper and Lower level of blockade |
| PACU Arrival | Date and Time |
| Pain scores @ rest: One hour after entry at PACU; average pain score during the nights as informed on the morning APS rounds; pain scores on each morning until discharge. | 0-10 (NRS) |
| Pain Score with Movement: One hour after entry at PACU; average pain score during the nights as informed on the morning APS rounds; pain scores on each morning until discharge. | 0-10 (NRS) |
| Rescue Analgesia onset | Date and Time Given |
| Hydromorphone or Morphine Consumption (PACU, 24 and 48 hrs Postoperatively) Morphine will be converted Mg of Hydromorphone for ease of comparison.  5 mg of Morphine is equivalent to 1 mg of Hydromorphone | Mg Hydromorphone |
| Opioids in PACU and up to 24 hrs since the end of surgery |  |
| From the first 24 hrs up to hospital discharge (excluding the above) |  |
| Total Volume Local Anesthetic infused | mls local anesthetic |
| Patient Satisfaction Score | 1 to 5 items, Likert scale |
| Adverse Events |  |

NRS: numerical rating scale; PACU: post-anesthetic care unit

**REFERENCES**

1 Bijur PE, Latimer CT, Gallagher EJ. Validation of a verbally administered numerical rating scale of acute pain for use in the emergency department. *Acad Emerg Med.* 2003;10:390–392.

2 Marcus DA, Bernstein C, Albrecht KL. Brief, Self-Report Fibromyalgia Screener Evaluated in a Sample of Chronic Pain Patients. *Pain Med.* 2013;14:730–735.
