# Supplementary material for: Continuous erector spinae plane block versus intercostal nerve block in patients undergoing video-assisted thoracoscopic surgery: a pilot randomized controlled trial
Source: Pilot Feasibility Stud. 2021 Feb 24;7:56. doi: 10.1186/s40814-021-00801-7 (PMC7903734; doi:10.1186/s40814-021-00801-7)
Supplement: Supplementary file 2 — Additional file 2: Appendix 2 Appendix_Calculation of group sample size. A supplementary document that presents the data for sample size estimates for the main trial (primary outcome, cumulative opioid consumption at 24 h). [file 40814_2021_801_MOESM2_ESM.doc]

Appendix 2

Calculation of group sample size for the main trial.

Observed estimates from pilot trial:

Minimum important difference = 1.63

SD of control group =4.17

SD of treatment group =2.22

| Minimum important difference | Control group  SD | Treatment group  SD | Sample size per group | Total sample size |
| --- | --- | --- | --- | --- |
| 1.50 | 4.17 | 3.50 | 104 | 208 |
| 1.63 | 4.17 | 3.50 | 88 | 176 |
| 2.00 | 4.17 | 3.50 | 59 | 118 |
| 2.50 | 4.17 | 3.50 | 38 | 76 |
| 3.00 | 4.17 | 3.50 | 26 | 52 |
| 1.50 | 4.17 | 2.22 | 78 | 156 |
| 1.63 | 4.17 | 2.22 | 66 | 132 |
| 2.00 | 4.17 | 2.22 | 44 | 88 |
| 2.50 | 4.17 | 2.22 | 29 | 58 |
| 3.00 | 4.17 | 2.22 | 20 | 40 |
| 1.50 | 4.17 | 1.50 | 69 | 138 |
| 1.63 | 4.17 | 1.50 | 59 | 118 |
| 2.00 | 4.17 | 1.50 | 39 | 78 |
| 2.50 | 4.17 | 1.50 | 25 | 50 |
| 3.00 | 4.17 | 1.50 | 18 | 36 |
| 1.50 | 3.50 | 2.22 | 60 | 120 |
| 1.63 | 3.50 | 2.22 | 51 | 102 |
| 2.00 | 3.50 | 2.22 | 34 | 68 |
| 2.50 | 3.50 | 2.22 | 22 | 44 |
| 3.00 | 3.50 | 2.22 | 15 | 30 |
